# Tirzepatide leads to weight reduction in people with obesity due to MC4R deficiency

**DOI:** 10.1038/s41591-025-03913-2

**Published:** 2025-08-26

**Authors:** Pallav Bhatnagar, Nadia N. Ahmad, Xuan Li, Matthew Coghlan, Lee M. Kaplan, I. Sadaf Farooqi

**Affiliations:** 1 Eli Lilly and Company, Indianapolis, IN USA; 2 Geisel School of Medicine at Dartmouth, Hanover, NH USA; 3 Institute of Metabolic Science and NIHR Cambridge Biomedical Research Centre, Addenbrooke’s Hospital, Cambridge, UK

**Keywords:** Metabolism, Endocrine system and metabolic diseases

## Abstract

The magnitude of weight reduction in the SURMOUNT-1 trial of the dual GLP-1 and GIP receptor agonist tirzepatide suggests that this treatment may be particularly effective in addressing the treatment needs of people with severe obesity (body mass index >40 kg m^−2^), some of whom may carry rare penetrant genetic variants. Here we investigated the clinical response of men and women in the SURMOUNT-1 trial who carried pathogenic mutations in the melanocortin 4 receptor (*MC4R*) gene, the most common genetic cause of obesity. We found that 32 of 2,291 people (1.4%) for whom data were available carried pathogenic *MC4R* mutations. At baseline, *MC4R* mutation carriers exhibited a higher body mass index compared with noncarriers (40 kg m^−2^ versus 38 kg m^−2^; *P* = 0.036). In the treatment arm, the weight loss trajectory over 72 weeks was comparable in both groups: 18.3% weight reduction in *MC4R* mutation carriers versus 19.9% in noncarriers. We conclude that tirzepatide is an effective treatment for the most common genetic subtype of obesity, MC4R deficiency.

## Abstract (web summary)

Analyzing data from the SURMOUNT-1 trial, the authors show that the weight loss effects of tirzepatide treatment are similar in carriers and noncarriers of *MC4R* pathogenic mutations.

## Main

The new generation of obesity medications that target the glucagon-like peptide-1 (GLP-1) receptor and/or the glucose-dependent insulinotropic polypeptide (GIP) receptor are transforming the clinical care of people with obesity and its complications^1^. In clinical trials, the dual GIP and GLP-1 receptor agonist tirzepatide has been shown to result in substantial weight reduction (~20%) in people with obesity, with and without type 2 diabetes^2,3^. This magnitude of weight reduction suggests that tirzepatide may be particularly beneficial in people with severe obesity (defined as a body mass index (BMI) >40 kg m^−2^), who have the highest burden of complications and highest mortality from cardiovascular disease.

Twin, family and adoption studies have consistently demonstrated that genetic factors influence the variation in body weight seen in an obesogenic environment^4^. The heritable contribution to body weight is greatest in people with severe obesity who have both a higher burden of common obesity susceptibility alleles and a higher prevalence of rare penetrant alleles that drive weight gain from childhood^5^. The melanocortin 4 receptor (MC4R) is expressed in the hypothalamus, brainstem and other brain regions and plays a pivotal role in the regulation of hunger, satiety and food preference^6^. Heterozygous *MC4R* mutations that are dominantly inherited and cause loss of function (LoF) in cells are defined as pathogenic^7,8^. Pathogenic *MC4R* mutations have been found in both clinical and population-based cohorts^9^ at varying frequencies depending on ascertainment criteria: 0.3% of an unselected UK birth cohort^10^, 1% of adults with a BMI >30 kg m^−2^, 2% of children with obesity^11^ and up to 5% of children with severe obesity^7^. MC4R deficiency, which represents the most common genetic form of obesity, is characterized by hyperphagia (increased drive to eat), weight gain that begins in the first 5 years of life, disproportionate hyperinsulinemia and accelerated linear growth in childhood^7,12,13^. Adults with MC4R deficiency often have severe obesity but with a lower prevalence of hypertension and reduced systolic and diastolic blood pressure associated with impaired sympathetic nervous system tone^14,15^.

Obesity due to MC4R deficiency is challenging to treat. Two studies have shown that intense dietary and physical activity interventions^11,16^ are less effective in children with MC4R deficiency, and weight reduction is harder to maintain in this group. While melanocortin receptor agonists can act as pharmacological chaperones to rescue signaling by some MC4R-mutant receptors in cells^17,18^, clinical trials of a MC4R agonist have not demonstrated efficacy in this patient group^17^. As such, there is currently no licensed treatment for people with obesity due to MC4R deficiency. GLP-1 receptor agonists predominantly target non-MC4R-dependent neural pathways in mice^19^, and there is evidence from a small clinical study with liraglutide^20^ that they may be effective in people with MC4R deficiency. Here, to investigate the efficacy of tirzepatide in people with MC4R deficiency, we examined genetic data obtained from participants in a randomized controlled trial (SURMOUNT-1) of people with a BMI ≥30 kg m^−2^, or ≥27 kg m^−2^ with at least one weight-related comorbidity^2^, treated with tirzepatide versus placebo.

DNA samples of sufficient quality were obtained on 2,291 people (90%) randomized in the SURMOUNT-1 trial. These samples were genotyped using the Axiom genotyping array from Affymetrix. We identified 24 different missense, frameshift and amino acid insertion or deletion mutations; 14 of these mutations have been shown to reduce MC4R function in cells (that is, LoF; https://www.mc4r.org.uk/), giving a prevalence of pathogenic *MC4R* mutations of 1.4% (*n* = 32/2,291) in adults with obesity in the SURMOUNT-1 study (Extended Data Table 1). People carrying either of the two prevalent gain-of-function *MC4R* variants (V103I and I251L; *n* = 88), associated with lower BMI in cohort studies^21^, were excluded from all analyses.

**Fig. 1:**
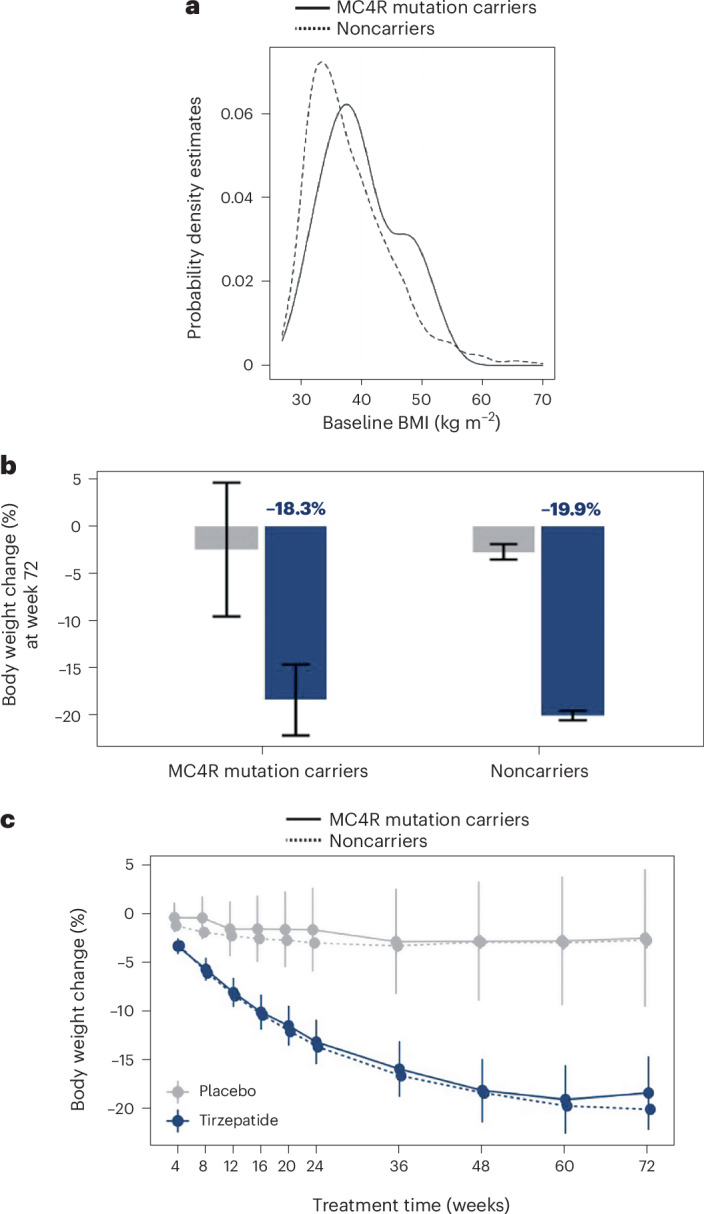
Baseline BMI distribution and tirzepatide-induced weight loss in *MC4R* mutation carriers versus noncarriers. **a**, The distribution of BMI at baseline for *MC4R* mutation carriers and noncarriers. **b**, Least-squares mean estimands of the percentage change in body weight from baseline to week 72, with error bars representing 95% confidence intervals (CI). Genetic effects on change in body weight were assessed using aggregated data on carriers of 14 *MC4R* LoF mutations (beta coefficient −0.88, SEM 3.2); statistical significance was assessed using a REGENIE collapsed burden test incorporating the treatment interaction effect (unadjusted two-sided *P* value 0.79). **c**, Time course effect on body weight change. Least-squares mean estimands were generated for each time point based on a three-way interaction among *MC4R* carrier status, pooled treatment and visit. Blue and gray bars represent tirzepatide treatment and placebo arms, respectively, with error bars representing 95% CI.

We found that, at baseline, the BMI distribution differed between *MC4R* mutation carriers and noncarriers (*P* = 0.036); a greater proportion of people with MC4R deficiency had severe obesity and increased waist circumference (*P* = 0.012); they were also slightly younger at recruitment (Table 1 and Fig. 1a). A trend toward lower total cholesterol was observed, consistent with findings from larger clinical case series of individuals with MC4R deficiency and from *MC4R* mutation carriers in the UK Biobank population cohort (accompanying paper, Zorn et al.^22^). Otherwise, the clinical characteristics of carriers of MC4R mutations and noncarriers were broadly similar (Table 1 and Extended Data Table 2).

**Table 1:** Characteristics of SURMOUNT-1 participants at baseline

Characteristic	*MC4R* mutation carriers (*n* = 32)^a^	Noncarriers (*n* = 2,259)^a^	*P* value^b^
Age, years	41.06 (13.12)	45.23 (12.36)	0.043
Female sex, no. (%)	20 (62%)	154 (68%)	
Duration of obesity, years	16.38 (9.17)	14.40 (10.88)	0.11
BMI, kg m^−2^	40.04 (6.21)	38.04 (6.88)	0.036
BMI category, no. (%)			0.084
<35	7 (22%)	907 (40%)	
≥35 to <40	10 (31%)	631 (28%)	
≥40	15 (47%)	721 (32%)	
Waist circumference, cm	120.44 (15.95)	114.02 (15.18)	0.012
Glycated hemoglobin, %	5.57 (0.48)	5.56 (0.37)	0.71
Fasting glucose, mg dl^−1^	95.20 (10.54)	95.50 (10.10)	0.72
Fasting insulin, mIU l^−1^	15.40 (9.08)	14.02 (10.44)	0.31
Blood pressure, mm Hg			
Systolic	121.31 (13.35)	123.59 (12.62)	0.43
Diastolic	79.5 (8.10)	79.56 (8.14)	0.91
Heart rate, beats min^−1^	72.50 (10.13)	72.13 (9.52)	0.83
Lipid levels, mg dl^−1^			
Total cholesterol	183.56 (36.94)	191.91 (38.94)	0.22
HDL cholesterol	45.87 (11.99)	48.96 (13.04)	0.23
LDL cholesterol	108.71 (33.35)	114.52 (32.81)	0.31
Triglycerides	148.14 (73.44)	145.80 (107.45)	0.52

^a^For all characteristics, mean (s.d.) are shown; no., number (%).

^b^Wilcoxon rank-sum test; Pearson’s chi-squared test; Fisher’s exact test, two-sided.

Lipid levels are shown as geometric means (coefficient of variation, %).

HDL, high-density lipoprotein; LDL, low-density lipoprotein.

We compared the weight loss trajectory at 72 weeks in the 32 *MC4R* mutation carriers and 2,259 noncarriers. People with MC4R deficiency responded to tirzepatide similarly to those with a normal *MC4R* genotype, demonstrating the effectiveness of tirzepatide in this subgroup of people with obesity (Fig. 1b,c). Furthermore, there was no differential impact of treatment (versus placebo) on metabolic parameters in *MC4R* mutation carriers versus noncarriers (Extended Data Table 3). All the *MC4R* mutation carriers in the placebo-treated group were female (Extended Data Table 2). This probably represents a chance occurrence given the rarity of MC4R deficiency and the fact that the SURMOUNT-1 cohort recruited twice as many females as males (males 825, females 1,714, total 2,539).

These results suggest that tirzepatide may be an effective treatment option for the most common form of monogenic obesity, MC4R deficiency. Given the severity of obesity in mutation carriers, equitable access to obesity medications should be a priority for this group, in whom diet and exercise interventions are unlikely to be effective. Roux-en-Y bypass surgery is effective in carriers of heterozygous but not homozygous mutations in *MC4R*, suggesting that central melanocortin circuits mediate some of the effects of bariatric surgery^23^. In a case report, a homozygous *MC4R* mutation carrier not responding to RYGB surgery was found to respond to GLP-1 receptor agonist treatment^24^.

Ongoing studies of the safety and efficacy of chronic treatment with obesity medications will be important for the management of patients with MC4R deficiency and other clinical groups. As MC4R deficiency presents with severe obesity from childhood, trials of tirzepatide (NCT06439277 and NCT06075667) and other obesity medications in children and adolescents with obesity, including those with MC4R deficiency, are needed to provide the evidence base for earlier, and potentially chronic, treatment in genetically driven obesity.

## Methods

### Study cohort (SURMOUNT-1 clinical trial)

#### Ethical approval and study populations

The study was conducted in accordance with the principles of the Declaration of Helsinki, Good Clinical Practice guidelines and all applicable regulatory requirements Study protocol and informed consent documents were approved by independent ethics committees and institutional review boards at all participating trial sites. Trial site investigators obtained consent, recruited participants, collected data and adhered to ethical standards. The sponsor conducted centralized monitoring with strict ethical oversight.

#### Genotyping

DNA was extracted from whole blood samples from participants enrolled in the SURMOUNT-1 trial (supported by Eli Lilly and Company.; ClinicalTrials.gov number NCT04184622) who consented to genetic analyses. Samples were quantified using a Quant-iT PicoGreen dsDNA assay to measure the DNA concentration. Samples were genotyped using the customized Axiom Biobank genotyping array (version 3) from Affymetrix. The array was customized using an Affymetrix UK BioBank backbone and incorporated all known genetic variants associated with genes or pathways related to various pathophysiological manifestations of obesity and related complications. A total of 2,474 patient samples along with duplicates and HapMap controls were genotyped, and data on 726,107 variants were generated. Standard metrics for genome-wide level data quality control were applied. After excluding variants with a call rate below 95% (*n* = 1,931), discordant variants in duplicate samples (*n* = 493) and monomorphic variants (*n* = 77,179), 646,504 variants and 2,291 samples passed quality control screening.

#### Statistical methods and details

##### Mixed-model repeated-measures weight loss imputation

A mixed model was run using SAS, including baseline weight, country, sex, prediabetes status, treatment, visit and the treatment-by-visit interaction as fixed effects, with a subject-level random effect. Any visits that did not have a recorded weight value were imputed as the predicted value from the mixed effect model. The value for the imputed weight percentage change from baseline was computed as (Predicted − Baseline) × 100/Baseline for all imputed visits. To ensure enough visits for imputation, we used only participants with a visit at week 24 or later (*n* = 2,291).

##### Genetic analysis

REGENIE was used to perform statistical association analyses for gene-level and single mutation-level testing^25^. For both models, the imputed weight loss percentage change from baseline at week 72 was used as the outcome. The model tested for genetic interaction with pooled treatment (collapsed 5, 10 and 15 mg treatment groups versus placebo), adjusting for age, sex, study country, baseline weight, prediabetes status and the first ten principal components.

$$\begin{array}{l}{{\mathrm{Outcome}}}={\beta}_{0}+{{\mathrm{Variant}}}\times {\beta }_{{{\mathrm{SNP}}}}+{{\mathrm{Treatment}}}\times {\beta }_{{{\mathrm{Trt}}}}+{{\mathrm{Variant}}}\\\qquad\qquad\quad\times\,{{\mathrm{Treatment}}}\times{\beta}_{{{\mathrm{inter}}}}+{{\mathrm{Covariates}}}\times {\beta }_{\mathrm{cov}}\end{array}$$

Default settings were used in REGENIE genotypic data preparation and testing. For gene-level MC4R carrier testing, 14 LoF mutations were used to define the MC4R mask for collapsed burden testing, which measures the genotype–treatment interaction effect.

##### Mixed-model repeated-measures least-square means

A mixed-effects model for the imputed weight outcome at week 72 was created, including the same fixed covariates as in the genetic analysis, as well as visit, and a subject-level random effect. The least-square means estimands were generated for the three-way interaction for MC4R carrier status, pooled treatment (collapsed 5, 10 and 15 mg treatment groups versus placebo) and visit. Week 72 visit estimands were plotted to visualize the single-time-point treatment and MC4R LoF carrier interaction effect (Fig. 1b), while estimands from all available visits were plotted over time to illustrate the visit interaction effect (Fig. 1c).

### Reporting summary

Further information on research design is available in the Nature Portfolio Reporting Summary linked to this article.

## Online content

Any methods, additional references, Nature Portfolio reporting summaries, source data, extended data, supplementary information, acknowledgements, peer review information; details of author contributions and competing interests; and statements of data and code availability are available at 10.1038/s41591-025-03913-2.

## Supplementary information

Reporting Summary

## Figures and Tables

**Extended Data Table 1:**
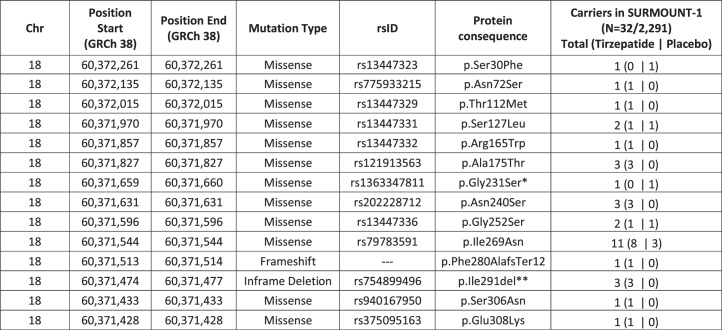
Summary of loss-of-function MC4R mutations identified in SURMOUNT-1 participants

* This is a multiallelic site (p.Gly231Ser and p.Gly231AlafsTer11); we only observed Gly231Ser in our study

** This is a multiallelic site (p.Ile291del, or p.Met292SerfsTer9, or p.Met292Ile); we observed p.Ile291del in our study.

MC4R variants were classified as loss-of-function if they had previously been shown to impair binding /signaling or other mechanisms in cells (as reported on www.mc4r.org.uk)

**Extended Data Table 2:**
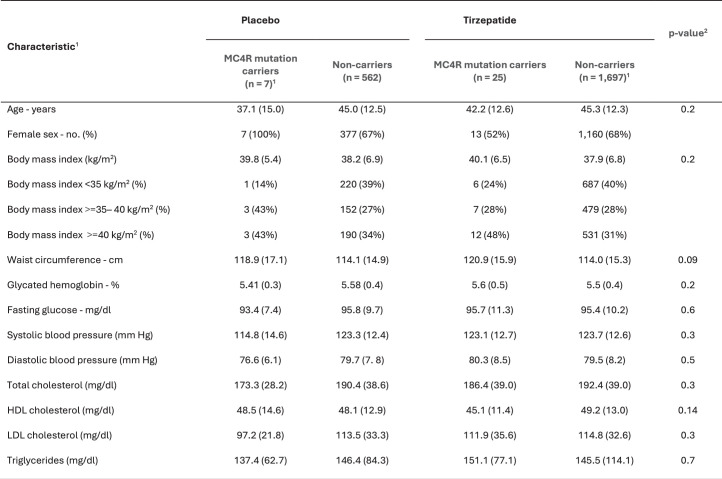
Characteristics of SURMOUNT-1 participants at baseline by treatment allocation

^1^For all characteristics, mean (SD) are shown; no, number (%); ^2^ Wilcoxon rank sum test; Pearson’s Chi-squared test; Fisher’s exact test, two-sided

Lipid levels are shown as geometric means (coefficient of variation, %). Abbreviations: HDL: High density lipoprotein; LDL: Low density lipoprotein.

**Extended Data Table 3:**
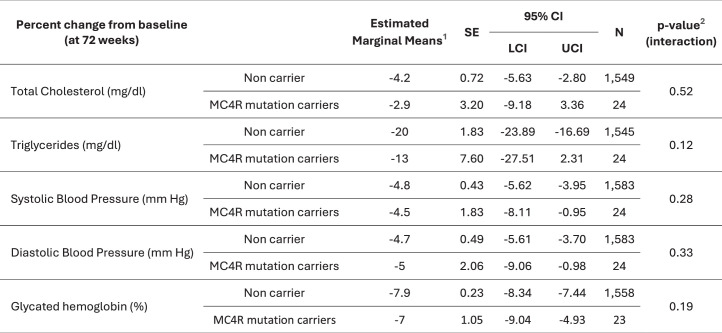
Tirzepatide induced changes in metabolic parameters

^1^Estimated marginal means are for the tirzepatide treatment arm only

^2^P-value represents MC4R mutation carrier status and treatment interaction effect from REGENIE collapsed burden test, two-sided, no multiple testing adjustment

N represents the participiants allocated to the tirzepatide treatment arm and with available MC4R genetic and clinical data

Total cholesterol and Triglycerides levels are shown as geometric means (coefficient of variation, %)

Abbreviations: SE: Standard Error for estimated marginal means; LCI: Lower bound 95% confidence interval; UCI: Upper bound 95% confidence interval

## Data Availability

The trial sponsor (Eli Lilly and Company) participated in the design and execution of the study, as well as collection, management, analysis and interpretation of the data. Patient-related information was anonymized and collected as part of the SURMOUNT-1 clinical trial and will be subject to confidentiality restrictions. Primary reasons for controlled access of this data are participant confidentiality and ethical compliance. Request for data access can be submitted via Vivli, and expected time for response is around 60 days.
