# Supplementary material for: Tirzepatide leads to weight reduction in people with obesity due to MC4R deficiency
Source: Nat Med. 2025 Aug 26;31(10):3294–6. doi: 10.1038/s41591-025-03913-2 (PMC12532586; doi:10.1038/s41591-025-03913-2)
Supplement: Supplementary file 1 — Reporting Summary [file 41591_2025_3913_MOESM1_ESM.pdf]

Corresponding author(s): Sadaf Farooqi

Last updated by author(s): May 19, 2025

Reporting Summary

Nature Portfolio wishes to improve the reproducibility of the work that we publish. This form provides structure for consistency and transparency in reporting. For further information on Nature Portfolio policies, see our Editorial Policies and the Editorial Policy Checklist.

Statistics

For all statistical analyses, confirm that the following items are present in the figure legend, table legend, main text, or Methods section.

n/a	Confirmed	
☐	☒	The exact sample size (n) for each experimental group/condition, given as a discrete number and unit of measurement
☐	☒	A statement on whether measurements were taken from distinct samples or whether the same sample was measured repeatedly
☐	☒	The statistical test(s) used AND whether they are one- or two-sided<br>*Only common tests should be described solely by name; describe more complex techniques in the Methods section.*
☐	☒	A description of all covariates tested
☐	☒	A description of any assumptions or corrections, such as tests of normality and adjustment for multiple comparisons
☐	☒	A full description of the statistical parameters including central tendency (e.g. means) or other basic estimates (e.g. regression coefficient) AND variation (e.g. standard deviation) or associated estimates of uncertainty (e.g. confidence intervals)
☐	☒	For null hypothesis testing, the test statistic (e.g. F, t, r) with confidence intervals, effect sizes, degrees of freedom and P value noted<br>*Give P values as exact values whenever suitable.*
☒	☐	For Bayesian analysis, information on the choice of priors and Markov chain Monte Carlo settings
☒	☐	For hierarchical and complex designs, identification of the appropriate level for tests and full reporting of outcomes
☐	☒	Estimates of effect sizes (e.g. Cohen's d, Pearson's r), indicating how they were calculated

Our web collection on statistics for biologists contains articles on many of the points above.

Software and code

Policy information about availability of computer code

Data collection	No software was used for data collection
Data analysis	SAS (Statistical Analysis System, software version 9.4) and R statistical software platforms were used for the analysis

For manuscripts utilizing custom algorithms or software that are central to the research but not yet described in published literature, software must be made available to editors and reviewers. We strongly encourage code deposition in a community repository (e.g. GitHub). See the Nature Portfolio guidelines for submitting code & software for further information.

Data

Policy information about availability of data

All manuscripts must include a data availability statement. This statement should provide the following information, where applicable:

- Accession codes, unique identifiers, or web links for publicly available datasets
- A description of any restrictions on data availability
- For clinical datasets or third party data, please ensure that the statement adheres to our policy

The sponsor (Eli Lilly & Co.) participated in the design and execution of the study, as well as in the collection, management, analysis, and interpretation of the data. Patient-related information was anonymized and collected as part of SURMOUNT-1 clinical trial and will be subject to confidentiality restrictions. Primary reasons for controlled access of this data are participant confidentiality and ethical compliance. Request for data access can be submitted via Vivli and expected time for response could be around 60-day review period.

Research involving human participants, their data, or biological material

Policy information about studies with human participants or human data. See also policy information about sex, gender (identity/presentation), and sexual orientation and race, ethnicity and racism.

Reporting on sex and gender	No
Reporting on race, ethnicity, or other socially relevant groupings	No
Population characteristics	Adults who were 18 years of age or older, with a body-mass index of 30 or more, or a BMI of 27 or more and at least one weight-related complication (e.g., hypertension, dyslipidemia, obstructive sleep apnea, or cardiovascular disease), and who reported one or more unsuccessful dietary effort to lose weight were eligible to participate.
Recruitment	Participant recruitment was done in Clinical trial setting and was approved by IRB at each trial site. All the participants provided written, informed consent before participation, and no potential biasness was introduced to participant recruitment and data analyses.
Ethics oversight	The study was conducted in accordance with the ethical principles of the Declaration of Helsinki, and Good Clinical Practice guidelines, and applicable regulatory requirements. Study protocol and informed consent documents were approved by independent ethics committees/institutional review boards (IRBs) at all participating trial sites. Trial site investigators obtained consent, recruited participants, collected data, and adhered to ethical standards. The sponsor conducted centralized monitoring with strict ethical oversight.

Note that full information on the approval of the study protocol must also be provided in the manuscript.

Field-specific reporting

Please select the one below that is the best fit for your research. If you are not sure, read the appropriate sections before making your selection.

☒ Life sciences ☐ Behavioural & social sciences ☐ Ecological, evolutionary & environmental sciences

For a reference copy of the document with all sections, see nature.com/documents/nr-reporting-summary-flat.pdf

Life sciences study design

All studies must disclose on these points even when the disclosure is negative.

Sample size	Out of the 2,474 total study samples, 2,291 samples with sufficient DNA quality and post-genetic QC were used for this study.
Data exclusions	183 samples were excluded due to standard quality control metrics for the genome-wide level data
Replication	Replication was not feasible due to the following justified reasons: (1) Genetic analyses was in a unique clinical trial context, (2) the original trial was powered to detect clinically meaningful treatment effects, (3) the original trial focused on drug-response relationship tied to the trial's therapeutic intervention. Replication would require identical drug regimens which are rarely available in independent datasets, and require prohibitively large resources.
Randomization	Participants were randomly assigned in a 1:1:1:1 ratio to receive tirzepatide at a dose of 5 mg, 10 mg, or 15 mg or placebo. For this genetic analyses we collapsed the treatment arms (i.e., 5, 10, 15 mg treatment groups vs. placebo)
Blinding	Double blinded

Reporting for specific materials, systems and methods

We require information from authors about some types of materials, experimental systems and methods used in many studies. Here, indicate whether each material, system or method listed is relevant to your study. If you are not sure if a list item applies to your research, read the appropriate section before selecting a response.

Materials & experimental systems

n/a	Involved in the study
☒	☐ Antibodies
☒	☐ Eukaryotic cell lines
☒	☐ Palaeontology and archaeology
☒	☐ Animals and other organisms
☐	☒ Clinical data
☒	☐ Dual use research of concern
☒	☐ Plants

Methods

n/a	Involved in the study
☒	☐ ChIP-seq
☒	☐ Flow cytometry
☒	☐ MRI-based neuroimaging

Clinical data

Policy information about clinical studies

All manuscripts should comply with the ICMJE guidelines for publication of clinical research and a completed CONSORT checklist must be included with all submissions.

Clinical trial registration	NCT04184622
Study protocol	Protocol for: Jastreboff AM, Aronne LJ, Ahmad NN, et al. Tirzepatide once weekly for the treatment of obesity. N Engl J Med 2022;387:205-16. (https://www.nejm.org/doi/suppl/10.1056/NEJMoa2206038/suppl_file/nejmoa2206038_protocol.pdf)
Data collection	All data was collected at specific clinics by investigators, with baseline defined as the last non-missing data collected at randomization (before the first dose of study drug) and post-treatment at 72 weeks. Data from a contracted vendor was stored electronically in the vendor's database system and then transferred to a centralized database.
Outcomes	Primary outcome: % change in body weight from baseline to week 72 (taken at fasted state using a calibrated electronic scale capable of measuring weight in kg to 1 decimal place)<br>Key secondary end points were metabolic parameters: waist circumference, HbA1c, fasting glucose, fasting insulin, BP, heart rate, and lipid levels

Plants

Seed stocks	N/A
Novel plant genotypes	N/A
Authentication	N/A
